# TLR4 predicts patient prognosis and immunotherapy efficacy in clear cell renal cell carcinoma

**DOI:** 10.7150/jca.84502

**Published:** 2023-07-16

**Authors:** Zhentao Zhang, Shuo Wang, Ye Lu, Demeng Xia, Ying Liu

**Affiliations:** 1 College of Basic Medicine, Naval Medical University, Shanghai 200433, China.; 2 Naval Hospital of Eastern Theater of PLA, Zhoushan, Zhejiang 316000, China.; 3 Department of Anesthesiology, Second Affiliated Hospital of Naval Medical University, Shanghai 200433, China.; 4 Department of Pharmacy, Seventh People's Hospital of Shanghai University of Traditional Chinese Medicine, Shanghai 200433, China.; 5 Institute of Translational Medicine, Shanghai University, Shanghai, 201900, China.

**Keywords:** Immunogenic cell death, TLR4, Clear cell Renal cell carcinoma, Immune cell infiltration

## Abstract

**Background:** Clear cell renal cell carcinoma (ccRCC) constitutes the commonest kidney malignancy. Immunogenic cell death (ICD) is a type of regulated cell death (RCD), which sufficiently activates adaptive immunity. However, ICD's involvement in cancer development is unclear, as well as the associations of ICD effectors with ccRCC prognosis.

**Methods:** RNA-sequencing expression profiles of ccRCC in The Cancer Genome Atlas (TCGA) and normal samples in Gene Expression Omnibus (GEO) were comprehensively investigated. Consensus clustering analysis was employed to determine subgroup members linked to ICD-related genes. Functional enrichment analysis was utilized for the examination of TLR4's biological role, and *in vitro* cellular assays were utilized for further confirmation. We also used Kaplan-Meier (KM) and Cox regression analyses to assess TLR4's prognostic value. Finally, “CIBERSORT” was employed for immune score evaluation.

**Results:** The associations of ICD effectors with ccRCC prognosis were examined based on TCGA, and 12 genes showed upregulation in ccRCC tissue specimens. Meanwhile, ccRCC cases with upregulated ICD-related genes had increased overall survival. Among these ICD-related genes, TLR4 was selected for subsequent analysis. TLR4 was upregulated in ccRCC samples and independently predicted ccRCC. TLR4 also enhanced the proliferative, migratory and invasive abilities in cultured ccRCC cells. Moreover, TLR4 had close relationships with immune checkpoints and infiltrated immune cells. ccRCC cases with elevated TLR4 expression had prolonged overall survival, suggesting a prognostic value for TLR4. Finally, a pan-cancer analysis demonstrated TLR4 had differential expression in various malignancies in comparison with normal tissue samples.

**Conclusions:** This study revealed prognostic values for ICD-associated genes, particularly TLR4, and experimentally validated the inducing effects of TLR4 on ccRCC progression *in vitro*. We also demonstrated the associations of TLR4 with immune cell infiltration, providing a novel strategy for prognostic evaluation and a novel therapeutic target in ccRCC.

## Introduction

Clear cell renal cell carcinoma (ccRCC), the commonest kidney malignancy, represents an aggressive malignancy arising from the proximal tubular epithelium and is associated with high mortality^1^, ^2^. Surgical or ablative strategies are the first-line treatment in ccRCC, in which successful treatment can be achieved in case of early detection. However, as many as one third of cases are metastatic at diagnosis or later develop metastasis. Metastatic ccRCC is generally fatal, with clear biological differences versus non-metastatic cancer, making it necessary to develop effective therapies for ccRCC^3^.

According to the Nomenclature Committee on Cell Death, immunogenic cell death (ICD) is classified as a regulated cell death (RCD) type sufficiently activating adaptive immunity in syngeneic individuals with immunocompetence. RCD's capability of driving adaptive immune response relies on antigenicity and adjuvanticity, and microbial composition also dramatically affect the predisposition of malignant cells to initiate adaptive immunity^4^. Cancer cells show pronounced antigenicity, at least partially resulting from tumor neoantigens (TNAs) and/or tumor-associated antigens (TAAs)^5^. Adjuvanticity mainly results from numerous damage‐associated molecular patterns (DAMPs) and cytokines emitted by malignant cells when they are in contact with RCD activators with potential immunogenicity. Mostly DAMPs and cytokines control immunostimulatory events through various pattern recognition receptors (PRRs), encompassing but not limited to Toll-like receptors (TLRs), RIG-I-like receptors, NOD-like receptors, Z-DNA binding protein 1, and heterogeneous nuclear ribonucleoprotein A2/B1^6^-^8^. Accumulating evidence demonstrates that the initiation of ICD represents an important treatment goal of cancer therapy, particularly considering the known success of immune checkpoint blockers^9^, ^10^. Besides, diverse ICD inducers are clinically successful, including anthracyclines, bleomycin, cyclophosphamide, dactinomycin and so on, which are associated with multiple DAMPs and ICD-associated cytokines^11^. Meanwhile, the practical value of ICD in the clinical context deserves further investigation. Therefore, identifying biomarkers that categorize patients, and exploring mechanisms for ICD-related genes contributing to tumor progression would be extremely beneficial.

TLRs as members of PRRs have critical functions in both innate and adaptive immune systems. TLRs could activate downstream pathways that trigger the production of multiple cytokines and chemokines and induce the maturation of immune cells^12^. TLR4 represents the firstly described homolog of the Drosophila Toll protein in humans, with high expression on immune (DCs, macrophages and lymphocytes), epithelial and malignant cells. The natural ligands of TLR4 include endotoxin (LPS) of Gram-negative bacteria, respiratory syncytial virus fusion protein and glucuronoxylomannan^13^. In addition to these natural ligands, multiple endogenous molecules, e.g., the heat shock protein family, extracellular matrix degradation products, high mobility group protein B1 (HMGB-1), β-defensin, and minimally modified LDL, could also activate TLR4 signaling pathways^14^. Abnormal TLR4 signaling induces cancer cell proliferation, accelerates cancer cell invasion and metastasis, protects cancer cells from apoptosis, and shapes a tumor-favoring cellular microenvironment^15^. Increased epithelial TLR4 signaling is also associated with inflammatory bowel disease (IBD) and colorectal tumorigenesis^16^. However, the role and clinical utilization of TLR4 in ccRCC are largely unexplored.

This work examined the associations of ICD-related genes, especially TLR4, with ccRCC prognosis via systematic and comprehensive bioinformatic analyses and experimental verification, providing novel insights into ccRCC treatment and prognosis.

## Materials and methods

### Data Collection and Processing

RNA-sequencing-based gene expression profiles for cancer and normal tissues were downloaded from a TCGA dataset (https://portal.gdc.cancer.gov/projects/TCGA-ccRCC) and Genotype-Tissue Expression (GTEx) data portal (https://www.gtexportal.org/home/datasets), respectively. GSE46699 and GSE 53757 were downloaded from the Gene Expression Omnibus (GEO) database (https://www.ncbi.nlm.nih.gov/geo/) to validate TLR4 expression patterns.

ICD-associated genes were retrieved from previously published reports^17^-^22^. TLR4 protein levels measured by immunohistochemistry in tumor and paired normal tissues were retrieved from Human Protein Atlas (https://www.proteinatlas.org/).

### Consensus Clustering

The “ConsensusClusterPlus” R package 1.54.0 was employed for consensus clustering to determine subgroup members associated with ICD-related genes. At most 6 clusters were involved, and 80% of all samples and genes were resampled for 100 times, clusterAlg=“hc”, distance="pearson".

A heatmap was generated for genes with SD>0.1. Samples from ccRCC patients downloaded from TCGA were grouped into 2 clusters depending on the expression of ICD-related genes. A cluster map was generated with the heatmap tool in R. The limma R package was used to assess differentially expressed mRNAs, based on adjusted p<0.05 and | log2FC| >2 between the 2 clusters.

### Functional Enrichment Analysis

Gene Ontology (GO) and Kyoto Encyclopedia of Genes and Genomes (KEGG) analyses were performed for the identification of differential signaling pathways and biological functions between the ICD-low- and -high groups. The “ClusterProfiler” package 3.18.0 in R was used for the identification of GO functions and KEGG pathways involving the differentially expressed genes (DEGs). Boxplots and heatmaps were generated with “ggplot2” and “heatmap” in R.

### Cell Culture

The human renal cell carcinoma 786-O (cat.no. FH0229) and Caki-1 (cat. no. FH0231) cell lines, provided by Fu Heng Cell Center (China), underwent culture in RPMI-1640 and McCoy's 5A medium (Gibco, USA), respectively, containing 10% fetal bovine serum (FBS, Gibco) and 1% penicillin/streptomycin cocktail (Beyotime, China). Both cell lines were maintained in a humid incubator at 37˚C with 5% CO_2_.

### RNA interference

TLR4 shRNA and control shRNA plasmids were purchased from Genomeditech (China). Oligonucleotides used for TLR4 shRNA are shRNA-TLR4-1, shRNA-TLR4-2, and shRNA-TLR4-3 (Table ​1). Cell transfection was carried out with Lipofectamine 2000 (Invitrogen, USA) as directed by the manufacturer, and knockdown efficiency was assessed by reverse transcription PCR (RT-PCR) and immunoblot.

### RT-PCR

Total RNA extraction utilized the RNAfast200 kit (Fastagen Biotech Company, China) as directed by the manufacturer. Reverse transcription was carried out with 1 μg of RNA with the PrimeScript RT reagent Kit (TaKaRa, China). RT-PCR was carried out in triplicate on a StepOnePlus^TM^ real time PCR system (Applied Biosystems, USA) with SYBR Premix Ex TaqTM PCR Kit (TaKaRa). The following primers were applied: TLR4 (F, 5′-CTGCAATGGATCAAGGACCA-3′ and R, 5′-TCCCACTCCAGGTAAGTGTT-3'); GAPDH (F, 5′-AGGTCGGTGTGAACGGATTTG-3′ and R, 5′-GGGGTCGTTGATGGCAACA-3′).

### Immunoblot

Cell samples underwent lysis with cell lysis buffer supplemented with protease inhibitors (Beyotime) on ice for 20 min. Protein quantitation applied the BCA protein assay kit (Beyotime). Equal amounts of total protein were resolved by 10% SDS-PAGE, followed by electro-transfer onto polyvinylidene difluoride (PVDF) membrane (Millipore, USA). Next, a 1-h blocking was performed with 5% nonfat milk, followed by successive incubations with anti-TLR4 primary (Cell Signaling Technology, USA; Cat^#^: 38519, 1:1000) and anti-rabbit secondary (Cell Signaling Technology, Cat^#^: 5151, 1:30000) antibodies at 4°C (overnight) and ambient (1 h), respectively. An Odyssey infrared imaging system (LI-COR Biotechnology, USA) was utilized for data acquisition. Quantitation utilized ImageJ (National Institutes of Health, USA), with β-actin for normalization.

### Cell proliferation and colony formation assays

Transfected cells seeded in a 96-well plate (2×10^3^cells/well) underwent incubation for 0, 24, 48, 72 and 96 h. Then, the CCK-8 reagent (Beyotime) was supplemented at 10 μL/well, followed by a 2-h incubation at 37˚C, and absorbance reading at 450 nm on a plate reader (BioTek Instruments, USA).

In the clonogenic assay, 10^3^ cells/well were seeded in a 6-well culture plate, for a 14-day growth with medium change at 3-day intervals. Then, fixation was carried out with 4% paraformaldehyde for 20 min, followed by a 15-min crystal violet (Beyotim) staining at room temperature. The plates were rinsed with PBS and left to air-dry followed by colony count.

### Wound healing migration assay

Transfected cells were seeded in a 12-well dish (6×10^4^ cells/well) to grow into a monolayer (90% confluence). Then, the monolayer was scratched with a 200 µL pipette, followed by a 24-h culture in the medium. Cells were imaged at 0 and 24 h post-scraping under a light microscope (Olympus, Japan).

### Transwell Assay

The Transwell assay were carried out with Transwell systems with 8-µm pores (Corning, USA). Transfected cells were seeded into the upper chamber at 5×10^5^ cells/well in FBS-free medium. Medium with 10% FBS was placed in the inferior compartment. Following a 24-h incubation at 37°C, fixation was carried out with 4% paraformaldehyde, followed by crystal violet (Beyotime) staining. Then stained cells were observed under a microscope (Olympus). Five randomly selected high-power fields/chamber were assessed.

### Survival Analysis

The Kaplan-Meier (KM) method was applied for evaluating the effects ICD-related genes on prognosis in the above two clusters utilizing the “ggsrisk”, “survival”, “survminer”, and “timeROC” packages in R. P values and hazard ratios (HRs) with 95% confidence intervals (CIs) were obtained by the log rank test and univariable cox proportional hazards regression analysis.

Next, a multivariable Cox regression analysis was carried out to determine risk scores based on age, gender, TNM stage of the primary tumor, clinical grade, and smoking status. A predictive model was built with the nomogram and calibration curves were further examined.

### Immune Score Analysis

The “CIBERSORT” was employed for immune score evaluation^23^, applying “ggplot2” and “pheatmap” in R 4.0.3.

### Statistical Analysis

Between-group comparisons utilized the Wilcox test. KM curves were assessed by the log rank test. Receiver operating characteristic (ROC) curve analysis with “timeROC” in R was performed to determine the values of diverse genes in predicting overall survival (OS). Univariable and multivariable cox regression analyses were employed for the identification of valuable clinical items for nomogram building. Data analysis utilized the R software, with p<0.05 indicating statistical significance.

## Results

### Differentially expressed ICD-related genes between ccRCC and cancerous biopsies

**Figure 1:**
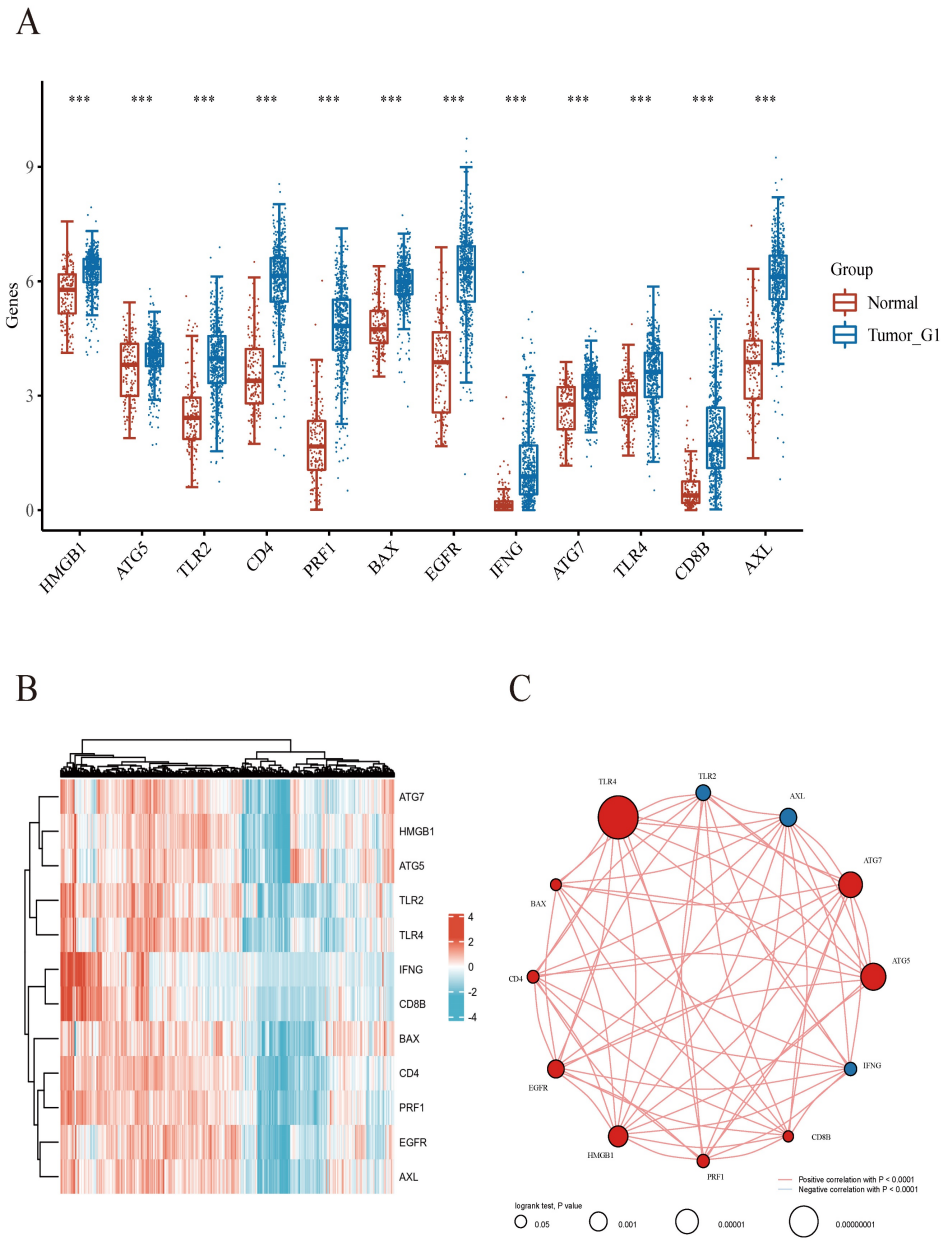
Comparison of gene expression for selected genes. **A** Abscissas and ordinates represent various sample groups and gene expression distributions, respectively. Red indicates normal tissue and blue represents tumor tissue. **B** Heatmap depicting the expression profiles of ICD-associated genes. **C**Spearman correlation and prognostic values of ICD-related genes in ccRCC. The circle size indicates the prognostic effect reflected by the p-value. The larger the circle the smaller log-rank p. Colors represent prognostic roles.

As reported previously, 12 genes (HMGB1, ATG5, TLR2, CD4, PRF1, BAX, EGFR, IFNG, ATG7, TLR4, CD8B, and AXL) were demonstrated to be associated with ICD^17^-^22^. Firstly, the expression patterns of the above 12 ICD-associated genes were comparatively assessed in ccRCC and normal tissue samples obtained from the TCGA and GTEx databases. Totally 72 ccRCC biopsies and 89 normal tissue biopsies were included in this analysis. The results showed that all these 12 ICD-related genes had differential expression between ccRCC and noncancerous tissue samples, with significant upregulation in ccRCC tissues (Figures 1A.B). Correlation analysis suggested 9 genes (HMGB1, ATG5, CD4, PRF1, BAX, EGFR, ATG7, TLR4, and CD8B) had positive associations among them (p<0.001), while 3 genes (TLR2, IFNG, and AXL) were negatively correlated with each other (p<0.001). Among them, TLR4 had the highest prognostic potential in ccRCC (Figure 1C).

### Consensus Clustering Analysis of ICD-Associated Genes revealed 2 ccRCC subgroups

**Figure 2:**
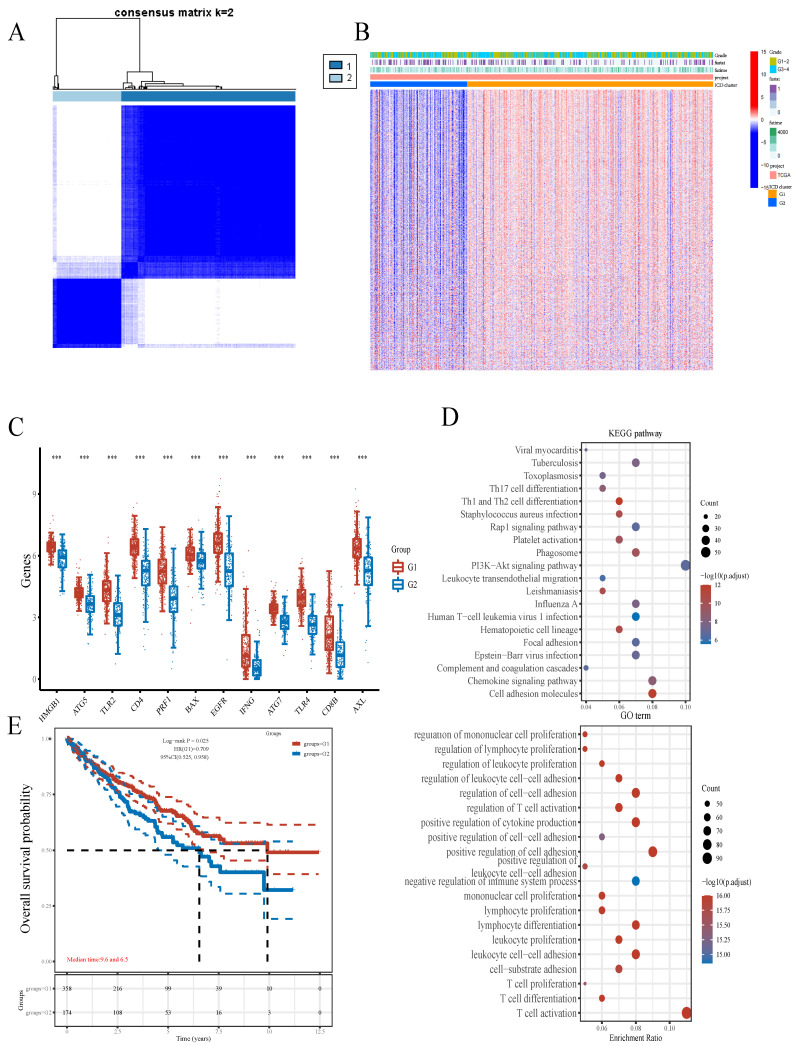
Comparative expression profiles of ICD-associated genes in the two ccRCC subclusters. **A** Consensus clustering matrix for k=2. **B** Expression heatmap for ICD-associated genes in various subgroups. Blue and red represent high and low expression levels, respectively. **C** Box plots indicate the expression of ICD-associated genes in the 2 subgroups G1 and G2. **D** GO and KEGG analyses of DEGs.

We next utilized the consensus clustering method to determine subgroup members of the ccRCC dataset in TCGA. Two distinct subgroups in the TCGA cohort were identified after k-means clustering (Figures 2A). Compared with subgroup G1, 8875 and 154 genes were downregulated and upregulated in G2, respectively (Figures 2B). In addition, all 12 included ICD-associated genes were upregulated in subgroup G1 compared with G2. Consequently, subgroup G1 was considered the ICD-high cluster, and subgroup G2 represented the ICD-low cluster (Figures 2C).

Then, we performed GO and KEGG analyses for further exploring the possible mechanisms by which ICD-related genes affect ccRCC progression. Through KEGG enrichment analysis, we found DEGs were enriched in PI3K-Akt signaling, Cell adhesion molecule, Chemokine pathway, Phagosome, Human T-cell leukemia virus 1 infection and so on. Meanwhile, GO analysis showed ICD-related genes were involved in T cell activation, positive regulation of cell adhesion, positive regulation of cytokine production, leukocyte cell-cell adhesion and regulation of cell-cell adhesion (Figure 2D). Besides, survival analyses showed different prognoses between the ICD-high and ICD-low groups (Figures 2E).

### TLR4 is upregulated in ccRCC samples

Previous studies revealed that TLR4 signaling is closely related to inflammation and cancer progression. For example, lipopolysaccharide (LPS), a well-known immune response inducer, activates TLR4 signaling to trigger pro-inflammatory reactions contributing to the eradication of invading microbes^24^. Besides, TLR4 promotes carcinogenesis through multiple inflammatory cytokines, including IL-6, IL-8 and TGF-β1. TLR4 overexpression also indicates poor prognosis in some cancers^25^.

**Figure 3:**
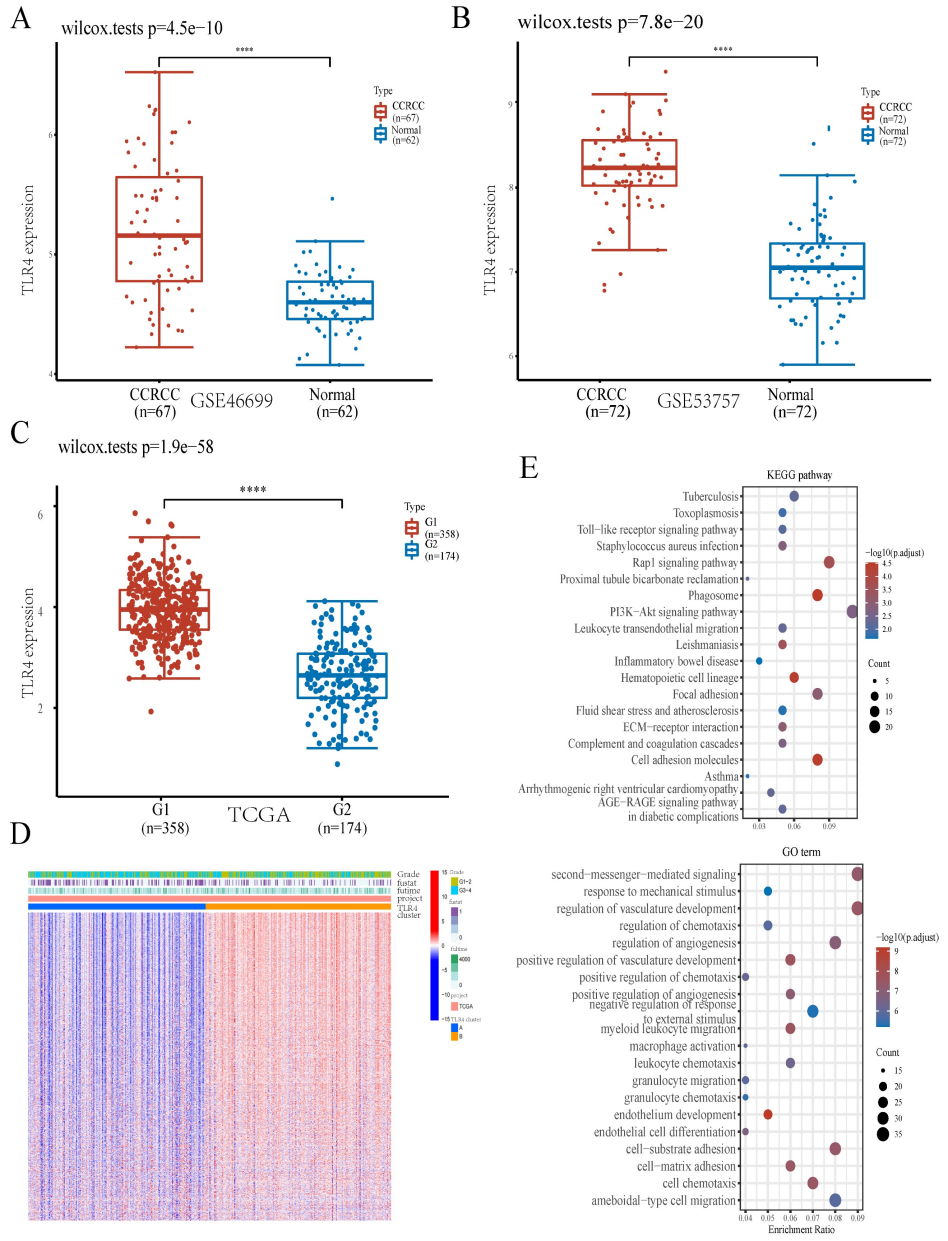
Comparative expression patterns of TLR4 in the two ccRCC subclusters. **A B** TLR4 expression in the GEO database.** C** TLR4 expression in TCGA. **D** Expression heatmap of TLR4 in various subgroups; red and blue represent high and low expression levels, respectively. **E** KEGG and GO analyses between the high- and low-TLR4 ccRCC cases.

In this work, TLR4 had the highest value in predicting prognosis in ccRCC (Figure 1C). In addition, TLR4 expression was elevated in subgroup G1 in comparison with subgroup G2 in TCGA (Figure 3C). We further verified TLR4's differential expression between ccRCC and noncancerous tissue specimens in another 2 datasets downloaded from the GEO database (GSE46699 and GSE53757), and found TLR4 mRNA amounts were markedly elevated in ccRCC compared with noncancerous tissue specimens (Figure 3A, B). TLR4 protein amounts were also markedly increased in ccRCC samples than noncancerous tissues as shown by immunohistochemistry data obtained from the Human Protein Atlas database (Figure S1B).

### Bioinformatics Analysis of TLR4

To obtain more insights into TLR4's role in ccRCC, ccRCC cases were subdivided into two clusters depending on TLR4 expression level. Compared with the TLR4-low cluster, there were 7692 upregulated (such as LYZ, MRC1 and NPR3) and 142 downregulated (such as SAA1, PPP1R1A and KRT19) genes in the TLR4-high cluster (Figures 3D). To confirm the mechanism of TLR4, we also performed GO and KEGG analyses. In KEGG analysis, TLR4 had tight associations with PI3K-Akt signaling, Rap1 signaling, Phagosome, Focal adhesion, Cell adhesion molecule and so on. GO analysis linked TLR4 with multiple molecular processes, including second-messenger-mediated signaling, regulation of vasculature development, regulation of angiogenesis, cell-substrate adhesion, ameboidal cell migration and so on (Figure 3E).

### Knockdown of TLR4 reduces the proliferative, invasive, and migratory abilities of ccRCC cells

**Figure 4:**
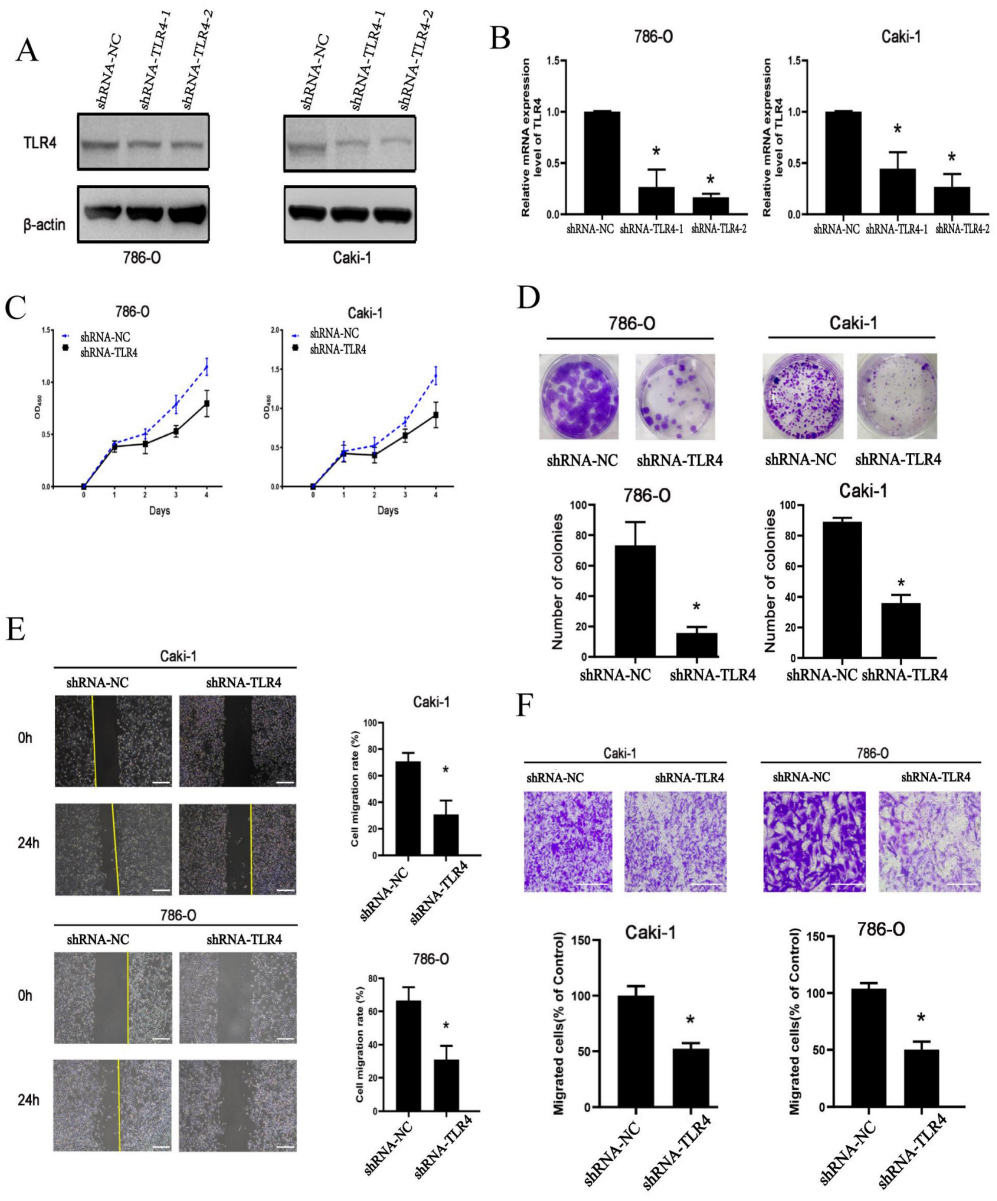
Cell proliferation, invasion, and migration in 786-O and Caki-1 cells following TLR4 knockdown. **A, B** Efficiency of TLR4 silencing in 786-O and Caki-1 cells detected by immunoblot and qPCR. **C** 786-O and Caki-1 cell proliferation assessed with CCK-8. **D** Representative images and quantitation of clonogenic assay in 786-O and Caki-1 cells. **E** Migratory capability of 786-O and Caki-1 cells detected by the wound healing assay; quantitation of scratched areas is shown in the right panel. **F** Representative images and quantitation for the transwell assay in 786-O and Caki-1 cells. Data are mean±SD from replicate wells. *P<0.05.

To determine TLR4's functions in ccRCC cells, loss-of-function assays were performed. Transfection with shRNAs targeting TLR4 for 72 h resulted in markedly reduced TLR4 mRNA and protein amounts in both 786-O and Caki-1 cells (Figure 4A and B). Proliferation ability was significantly decreased in both cell lines after transfection with TLR4-shRNA (Figure 4C). The clonogenic assay similarly demonstrated TLR4 suppression reduced proliferation in both cell lines (Figure 4D).

Next, the migratory and invasive capabilities of cells were examined. The scratch assay demonstrated migration rate was significantly reduced in both cell lines following TLR4-shRNA transfection compared with negative control cells at 24 h (Figure 4E). Furthermore, knockdown of TLR4 remarkably reduced the number of invasive ccRCC cells in the Transwell assay (Figure 4F). The above findings suggested knockdown of TLR4 inhibited the proliferative, invasive, and migratory abilities of ccRCC cells.

### Prognostic Value of TLR4 in ccRCC

**Figure 5:**
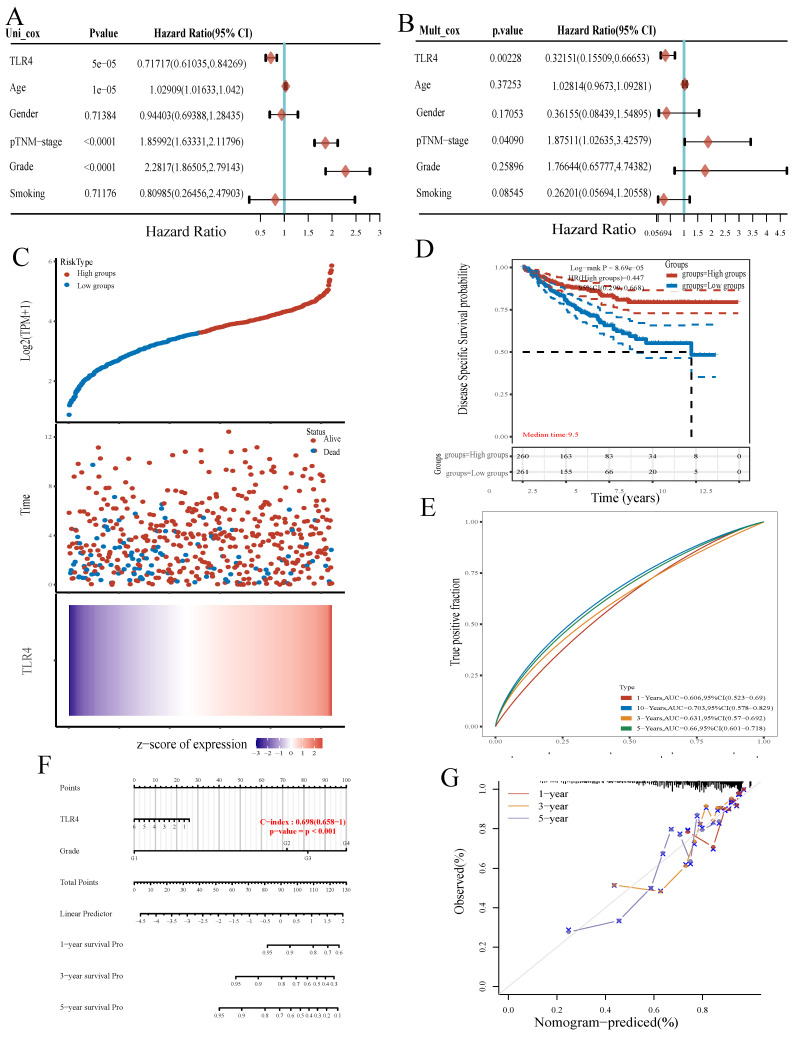
TLR4 expression is associated with prognosis. **A, B** Univariable and multivariable Cox regression analyses. **C** Expression distribution, survival analysis and survival status of TLR4 in the TCGA dataset. **D** Survival analysis of the gene signature from the TCGA dataset by the Kaplan-Meier method. **E** ROC curve analysis of the TCGA dataset for survival prediction. **F** Nomogram for prediction of OS in patients with ccRCC. **G** Calibration curves for the novel nomogram.

We further evaluated TLR4's prognostic value in ccRCC. Univariable analysis revealed TLR4 expression (HR=0.71717), age (HR=1.02909), p-TNM stage (HR=1.85992) and grade (HR=2.2817) had significant associations with OS in ccRCC (all p<0.001, Figure 5A). Multivariable analysis further demonstrated TLR4 expression independently predicted ccRCC progression (HR=0.32151, p<0.01, Figure 5B).

The ccRCC samples retrieved from TCGA were divided into the TLR4-high and TLR4-low groups depending on TLR4 expression. More patients were alive in the TLR4-high subgroup in comparison with the TLR4-low group (Figure 5C). In addition, ccRCC cases with elevated TLR4 also had longer OS in comparison with the low-TLR4 group (Figure 5D). Next, TLR4's efficiency for predicting prognosis in ccRCC was assessed by ROC curve analysis. The areas under the ROC curves (AUCs) for predicting 3-, 5, and 10-year survival were 0.631, 0.66, and 0.703, respectively (Figure 5E).

A novel nomogram with clinical usefulness was built, which was complementary to TLR4 expression (Figure 5F). The built nomogram predicted 1, 3 and 5-year OS rates relatively well (Figure 5G).

### Correlations of TLR4 with immune checkpoints and infiltrated immune cells

**Figure 6:**
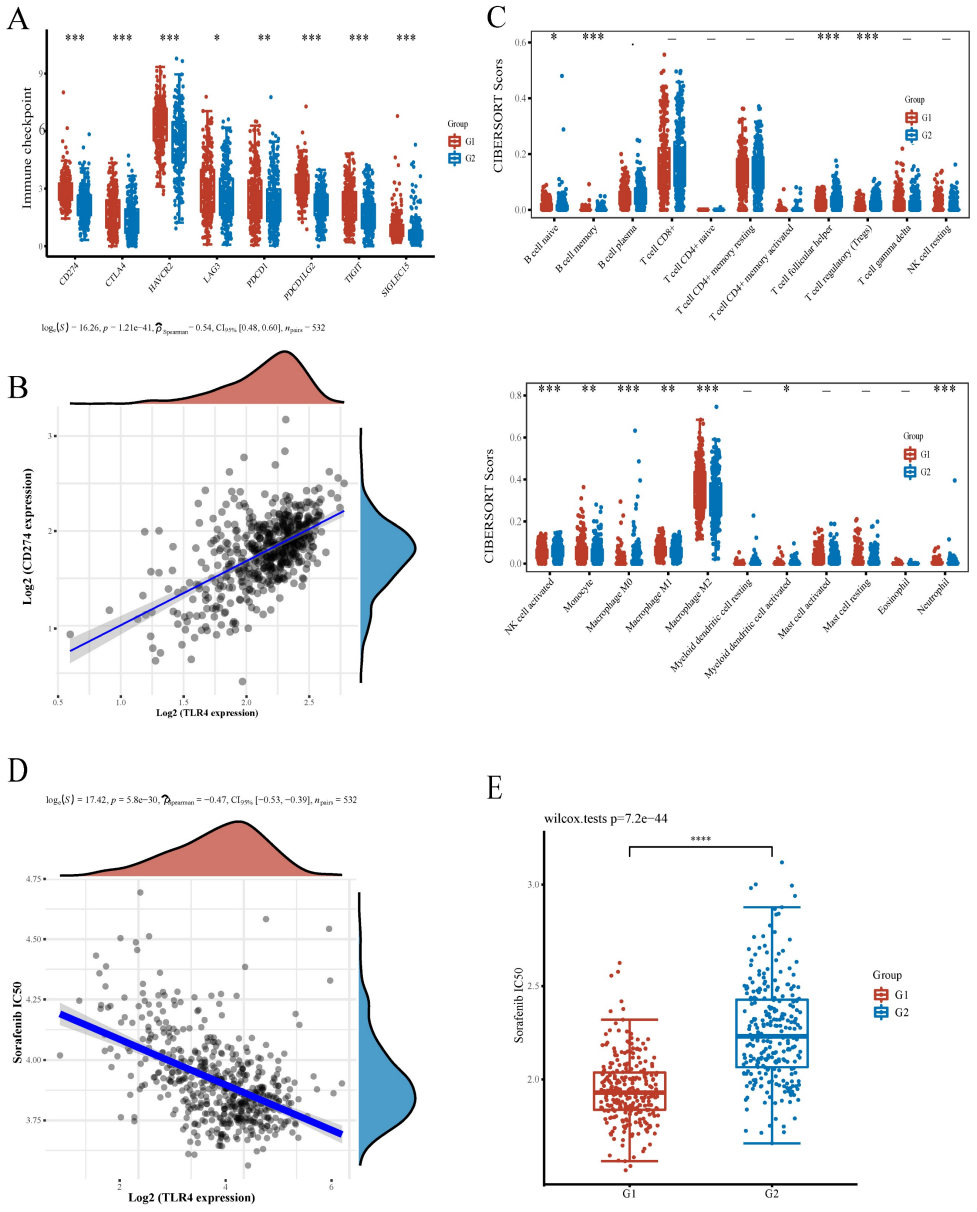
Associations of TLR4 with immunotherapy and infiltrating immune cells in ccRCC. **A** Expression of common immune checkpoints in the high- and low-TLR4 expression subgroups. **B** Association of PD-L1 with TLR4 in ccRCC. **C** Infiltrated immune cells in the high- and low-TLR4 groups. **D** Spearman correlation analysis of IC50 score and TLR4 expression. **E** IC50 score distribution in the high- and low-TLR4 expression groups.

To support the application prospect of TLR4 in immunotherapy, we evaluated the associations of TLR4 with immune checkpoints and infiltrated immune cells. Multiple immune checkpoints had significant differences between the high- and low- groups, e.g., CD274 (PD-L1), CTLA4, HAVCR2, LAG3, PDCD1 (PD-1), PDCD1LG2, TIGIT and SIGLECI5, providing potential therapeutic targets for ICB in ccRCC (Figure 6A). Immune checkpoint inhibitors interacting with programmed cell death 1 receptor (PD-1) are known to increase survival in a subset of ccRCC cases. However, PD-L1 expression is also increased on malignant cells, promoting immune escape^26^, ^27^. A positive association of TLR4 with PD-L1 in ccRCC was confirmed by Spearman correlation analysis (Figure 6B). Besides immune checkpoints, the associations of TLR4 with infiltrated immune cells in ccRCC cases were examined. Compared with the TLR4-low subgroup, the infiltration degrees of activated natural killer (NK) cells, follicular helper T (Tfh) cells, regulatory T cells (Tregs), memory B cells, M0 Macrophages and activated myeloid dendritic cells were significantly lower in the TLR4-high subgroup, while naïve B cells, monocytes, M1 macrophages, M2 macrophages and neutrophils had significantly higher infiltration degrees in the TLR4-high subgroup (Figure 6C).

Sorafenib represents the firstly developed targeted multi-kinase inhibitor and the first-line chemotherapeutic drug approved for RCC treatment^28^. To evaluate the potential role of TLR4 in chemotherapy, we estimated the IC50 based on the predictive model of sorafenib. A significant negative correlation was observed between TLR4 expression and the IC50 of sorafenib (Figure 6D). Subsequently, the differential IC50 of sorafenib between the two subgroups further suggested that the TLR4-high group was more sensitive (Figure 6E).

### Comprehensive Analysis of LIPT1 in Pan-Cancer

**Figure 7:**
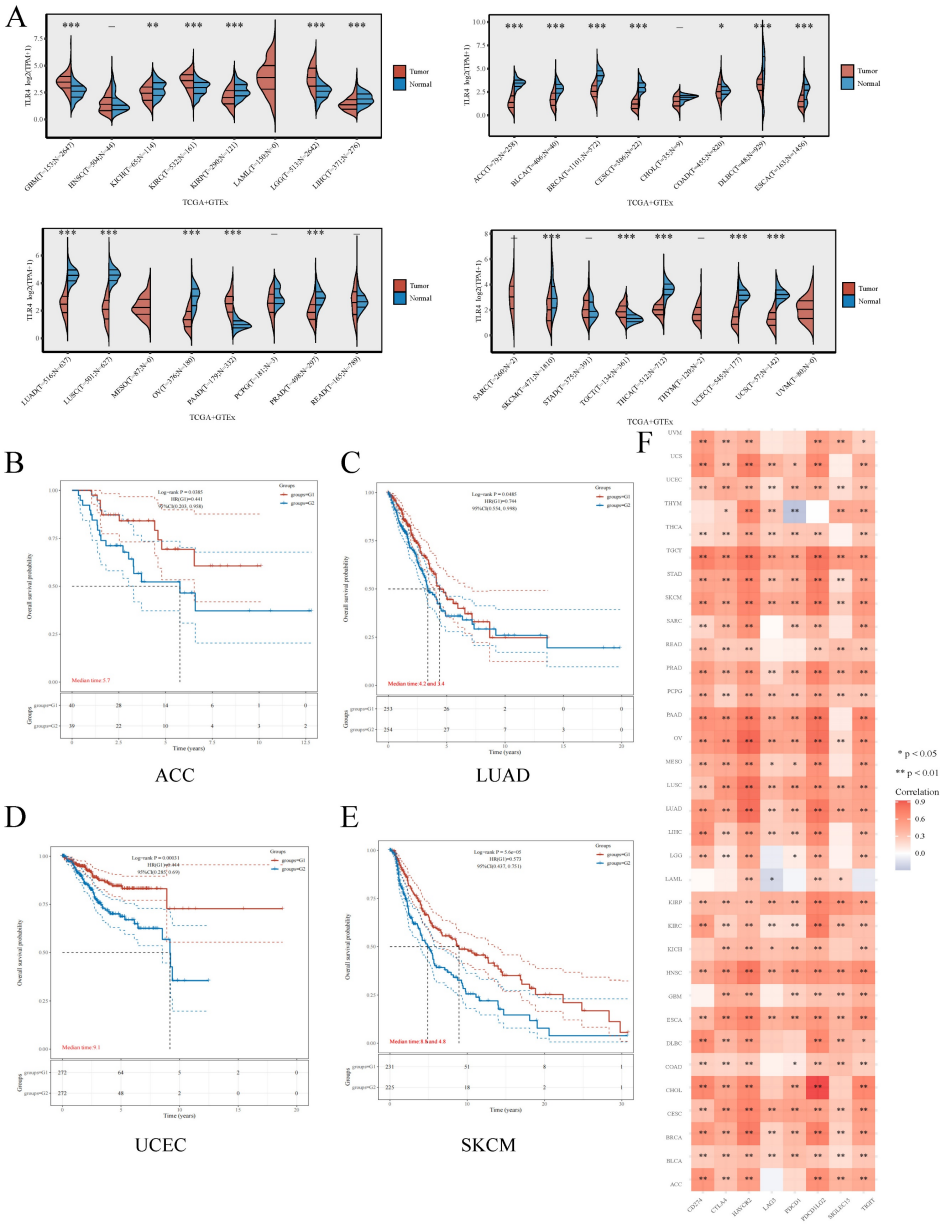
Comprehensive analysis of TLR4 in pan-cancers **A** Expression distribution of TLR4 in cancer and noncancerous tissue specimens in pan-cancer data of TCGA and GTEx. **B - E** Kaplan-Meier survival analysis of the high- and low-TLR4 groups in multiple cancers. **F** Expression heatmap of immune-checkpoint-associated genes. Each box corresponds to the correlation between TLR4 expression and an immune checkpoint in respective tumor samples.

Besides ccRCC, we also examined whether TLR4 expression has a potential in predicting prognosis in other cancers. For this purpose, TLR4 mRNA amounts were compared in multiple malignancies and the respective noncancerous tissue samples retrieved from TCGA and GTEx, respectively. Compared with normal tissues, TLR4 was upregulated in multiple cancers (GBM, KIRC, LGG and PAAD), and downregulated in ACC, BLCA, BRCA, COAD, CESC, DLBC, ESCA, KIRP, LIHC, LUAD, LUSC, OV, PRAD, THCA, UCEC and USE (Figure 7A). As in the above study, we also divided the tumor patients into the TLR4-high and TLR4-low subgroups for different types of tumors. TLR4-high cases also had prolonged OS in ACC, LUAD, UCEC and SKCM compared with the TLR4-low groups (Figure 7B, C, D, E). We next examined the associations of TLR4 expression with 8 immune checkpoints (PD-L1, CTLA4, HAVCR2, LAG3, PDCD1, PDCD1LG2, SIGLEC15, and TIGIT), and TLR4 expression was widely associated with the 8 immune checkpoints in most cancer types (Figure 7F).

## Discussion

Recent studies have described ccRCC as one of the cancers with highest immune and vascular infiltration rates, with immune checkpoint blockade (ICB) treatment and combination regimens markedly increasing patient survival in ccRCC^29^. So, predicting patients who may benefit from treatment is of great importance. ICD constitutes a form of cancer cell death, which could be induced by some antitumor therapeutic measures. Besides direct cancer cell killing by antitumor therapy, dying cancer cells release DAMPs, which induce tumor immunity and subsequently elicit long‐term efficacy of chemotherapeutics^19^, ^30^. Understanding the molecular pathways of ICD in ccRCC may help improve antitumor strategies. However, ICD's function in ccRCC remains largely unknown. The present work comprehensively assessed the expression profile of ICD-associated genes in ccRCC. As depicted above, all the 12 included ICD-related genes had significant differences between ccRCC and noncancerous tissue samples. Besides, the ICD-related genes detected in the current work had a good capability of distinguishing ccRCC from noncancerous specimens (Figure 1A, 1B), which strongly suggests a role for ICD in ccRCC diagnosis. Considering the various molecular profiles in ccRCC, we also performed consensus clustering analysis, which aims to aggregate the distinct clusters detected in a given dataset to obtain a better clustering solution^31^, to determine biological alterations in ccRCC involving ICD-related genes. Interestingly, ccRCC cases in the ICD-high cluster had substantially better prognosis (Figure 2A-2C, Figure 2E), which also suggested a role for ICD in the prediction of ccRCC prognosis.

ICD's capability of inducing adaptive immunity involves multiple factors, including the release of DAMPs from dying or injured cancer cells, antigen-presenting cell (APC) recruitment and maturation and cytotoxic T lymphocyte (CTL)-driven immunity^32^. Dendritic cells (DCs) have the features of antigen presentation and T-cell response stimulation. DCs strongly activate CD8+ cytotoxic T lymphocytes and CD4+ Th1 cells, conferring them critical roles in antitumor immunity^33^. Previously reported findings demonstrated TLR4 expressed on DCs is important in DC activation, and consequently enhances antitumor T-cell responses elicited by DAMPs from chemotherapy-treated malignant cells, which illustrates an essential role for TLR4 in ICD^34^. Besides, TLR4 is also expressed in multiple cell types, and numerous studies have demonstrated multiple roles for TLR4 in tumorigenesis. TLR4 is frequently upregulated in inflammatory bowel disease (IBD) and colorectal cancer (CRC). Induction of epithelial TLR4 contributes to dual oxidase 2 (DUOX2) in colitis, which drives the epithelial production of reactive oxygen species (ROS) and promotes the development of colitis-related tumors^16^. TLR4 also plays a key role in hyaluronic acid (HA) promotion of colon tumorigenesis^35^. It is well accepted that TLRs are PRRs that recognize “danger signals” to provoke an immune response. Danger signals include pathogen-associated molecular patterns (PAMPs) and DAMPs^36^. TLR4, as a receptor of resistin, which has high expression in human breast cancer, also mediates the promoting effect of resistin on epithelial-to-mesenchymal transition and stemness in breast cancer, combined with the downstream NF-κB/STAT3 pathway^37^. During inflammation-to-tumor transition, chronic infection induces TLR4 signaling, which triggers chronic inflammatory microenvironment and promotes cancer progression. *Helicobacter pylori* and hepatitis virus infections could activate TLR4 and increase TLR4 expression, which leads to gastric and liver cancer^38^.

Among ICD-associated genes, TLR4 had the highest prognostic value in ccRCC in the present study (Figure 1C). To obtain further insights into the involvement of TLR4 in ccRCC, bioinformatic and experimental analyses were carried out (Figure 3, 4). We found that TLR4 was upregulated in ccRCC samples at both gene expression and protein levels, and TLR4 knockdown inhibited the proliferative, invasive, and migratory abilities of ccRCC cells, which shows potential for providing a new strategy for ccRCC patients. From the mechanistic perspective, the most enriched KEGG pathway was the PI3-Akt signaling pathway in this study. Previous studies have also shown an overall gene regulation rate for 20 representative PI3K/AKT pathway effectors of 27.7% in the TCGA-ccRCC dataset^39^. Numerous studies have confirmed the PI3K/AKT pathway mediates multiple biological functions in ccRCC progression and metastasis^40^, ^41^, together suggesting a critical role for PI3K/AKT signaling and providing potential treatment opportunities.

After verifying TLR4's biological function in ccRCC, TLR4 was shown to independently predict ccRCC in terms of overall survival in TCGA. Both univariable and multivariable analyses suggested a prognostic value for TLR4 in ccRCC (Figure 5A, B). Despite the biological potential of TLR4 to promote tumor progression (Figure 4), the TLR4-high group had improved overall survival compared with the TLR4-low group (Figure 5C). Meanwhile, TLR4 is negatively correlated with the IC50 of sorafenib (Figure 6D, E), suggesting cases with elevated TLR4 expression have improved response to treatment. Due to the discrepancy between experimental results and real-world applications, the concept of real-world evidence (RWE) has attracted increasing attention^42^, ^43^. Treated patients were included in the calculation of overall survival, which suggests the value of real-world research on TLR4 in predicting overall survival. Of course, larger trials are warranted, and the accurate role of TLR4 in chemotherapy also needs further exploration. In addition, nomograms, consisting of numerous clinic indicators, are convenient and reliable tools for individual assessments and clinical decision making^44^. In this study, a nomogram combining TLR4 and clinical indicators was constructed for predicting patient prognosis in ccRCC patients. Scores for various indicators were added to estimate overall survival rate in ccRCC cases (Figure 5D, E). Taken together, ccRCC cases with elevated TLR4 expression might have better prognosis.

Immune checkpoint blockade (ICB)-based immunotherapeutic targeting cytotoxic T lymphocyte antigen 4 (CTLA4) or programmed cell death 1 (PD1) signaling have considerably improved ccRCC treatment. However, only some case obtain clinical benefits^45^. A previous study suggested PD-L1 upregulation on both tumor (TCs) and tumor-infiltrating immune (TICs) cells might improve patient response to immune checkpoint inhibitors (CPIs)^46^. A recent study showed TLR4 is positively correlated with programmed death ligand-1 (PD-L1) in human PDAC tissue samples, a correlation mediated by the TLR4 ligand LPS. LPS activates the canonical TLR4/MyD88/AKT/NF-κB pathway to upregulate PD-L1 transcription in cultured cells and animal models^47^. Similarly, TLR4 is mechanistically related to PD-L1 upregulation in non-small cell lung cancer^48^. As shown above, TLR4 and PD-L1 were positively correlated (Figure 6A, B), which may guide future development of immunotherapies in ccRCC.

ccRCC is characterized by a high extent of infiltrated immune cells , e.g., CD8 + T cells, CD4+ T cells, NKs and macrophages^49^. Immune cell infiltration would be useful for the assessment of clinical prognosis in patients with ccRCC. Among infiltrated immune cells, tumor-associated macrophages (TAMs) have great phenotypic and functional diversities. TAMs have been shown to contribute to tumor development through a variety of mechanisms. In addition, TAMs are considered to reflect clinical outcome in ccRCC^50^, ^51^. In this work, we also found that the expression of TLR4 is widely associated with the extent of macrophage infiltration (Figure 6C). Both M1 and M2 macrophages showed differential infiltration. Activated NK cells and neutrophils also showed differential infiltration, both of which are associated with patient prognosis in ccRCC^52^, ^53^. Taken together, besides adaptive immune response, innate immunity plays important roles in ccRCC patient outcome. Considering the importance of predicting the patients who would benefit from immune therapy, we also explored the correlations of TLR4 with immune checkpoints and infiltrated immune cells. Immune checkpoint gene expression was positively correlated with macrophage infiltration. This may be related to immune cell infiltration (mainly macrophages) and differential expression of immune checkpoint-related molecules. In addition, the TLR4-low subgroup group displayed higher levels of NK cell infiltration. Of course, the specific relationship between the two needed further exploration and experimental confirmation. The effects of TLR4 on immune cell infiltration and immunotherapy in ccRCC require experimental and clinical validation.

Although we showed TLR4 represents a potential biomarker of ccRCC, this study had limitations. First, despite a comprehensive search of publicly available databases for ccRCC, the sample size was limited, and larger trials are warranted. Secondly, for future validation of TLR4 in predicting patient prognosis, a prospective study should be conducted. Thirdly, further assays are required to precisely unveil the mechanism by which TLR4 affects ccRCC progression and treatment.

## Conclusions

Overall, all 12 ICD-associated genes were upregulated in ccRCC, and showed high efficiency in discriminating ccRCC and noncancerous samples. Among the ICD-associated genes, TLR4 had the highest predictive value for prognosis, and also enhanced the proliferative, migratory and invasive abilities of cultured ccRCC cells. A TLR4-related risk scoring system was generated, and could independently predict patient prognosis in ccRCC. Furthermore, TLR4 had close relationships with immune checkpoints and immune cell infiltration, suggesting that TLR4 gene expression may be a useful prognostic marker in ccRCC.

## Supplementary Material

Supplementary figures and tables, data.

## Figures and Tables

**Table 1:** Oligonucleotide sequences

Primer name	Sequence
shRNA-TLR4-NC	F:5′-GATCTGTTCTCCGAACGTGTCACGTTTCAAGAGAACGTGAC ACGTTCGGAGAATTTTTTC-3′
	R:5′-AATTGAAAAAATTCTCCGAACGTGTCACGTTCTCTTGAAAC GTGACACGTTCGGAGAACA-3′
shRNA-TLR4-1	F:5′-GATCCGCTTCATAAGCTGACTTTAAGTTCAAGAGACTTAAA GTCAGCTTATGAAGCTTTTTTG-3′
	R:5′-AATTCAAAAAAGCTTCATAAGCTGACTTTAAGTCTCTTGAA CTTAAAGTCAGCTTATGAAGCG-3′
shRNA-TLR4-2	F:5′-GATCCGCCTTTGTTATCTACTCAAGCTTCAAGAGAGCTTGA GTAGATAACAAAGGCTTTTTTG-3′
	R:5′-AATTCAAAAAAGCCTTTGTTATCTACTCAAGCTCTCTTGAA GCTTGAGTPAGATAACAAAGGCG-3′
shRNA-TLR4-3	F:5′-GATCCGCAGTCGTGCTGGTATCATCTTTCAAGAGAAGATGA TACCAGCACGACTGCTTTTTTG-3′
	R:5′-AATTCAAAAAAGCAGTCGTGCTGGTATCATCTTCTCTTGAA AGATGATACCAGCACGACTGCG-3′

## Abbreviations

ccRCC: Clear cell Renal cell carcinoma
ICD: Immunogenic cell death
RCD: regulated cell death
DEGs: Differentially expressed genes
TCGA: The Cancer Genome Atlas
GTEx: Genotype-Tissue Expression
GSVA: Gene Set Variation Analysis
PCA: Principal component analysis
