# Supplementary material for: TLR4 predicts patient prognosis and immunotherapy efficacy in clear cell renal cell carcinoma
Source: J Cancer. 2023 Jul 16;14(12):2181–97. doi: 10.7150/jca.84502 (PMC10414050; doi:10.7150/jca.84502)
Supplement: Supplementary file 1 — Supplementary figures and tables, data. [file jcav14p2181s1.zip › Figure S1-S2 .pdf]

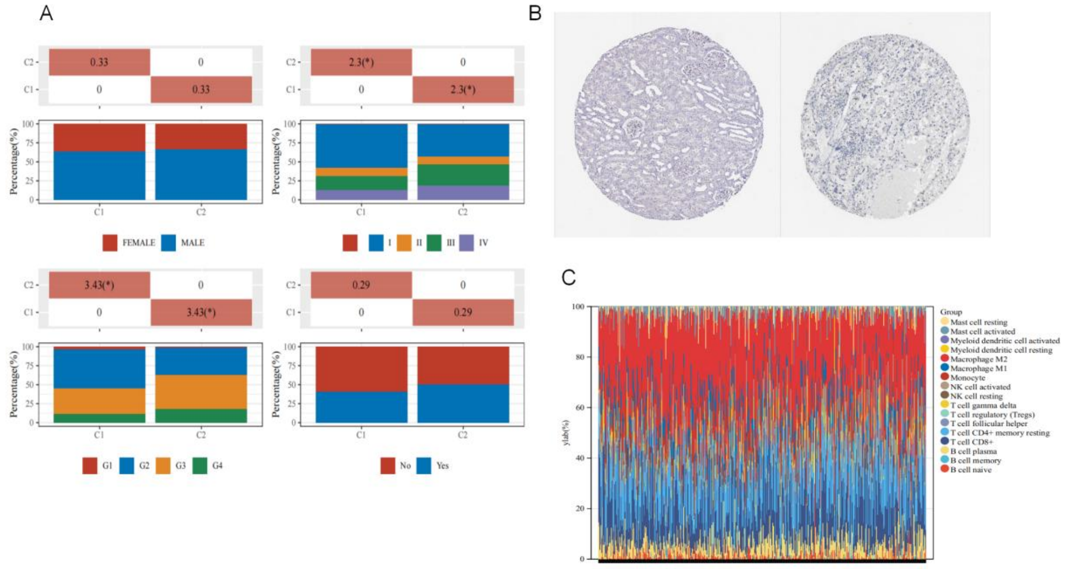

Figure S1 The clinical trait relationships of TLR4 of ccRcc patients.(A) The distribution of clinical characteristics in the samples in the high and low TLR4 expression groups. (B) TLR4 was highly expressed in ccRcc tissue (right) compared with normal skin tissue (left) in the human protein atlas based on immunohistochemistry analysis. (C) The percentage abundance of tumor-infifiltrating immune cells showed the immune infifiltration analysis between high and low TLR4 expression groups.

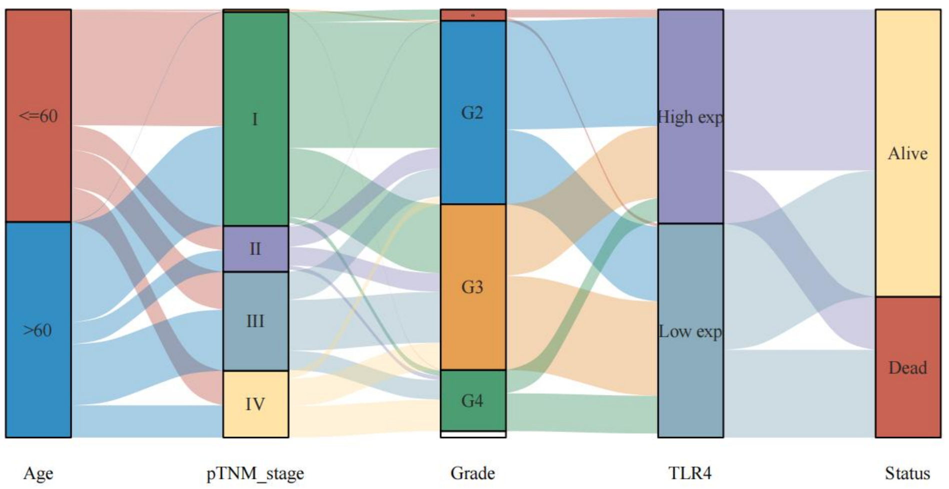

Figure S2 Sankey diagram demonstrate the trend of high and low gene expression distribution of TLR4 for different clinical characteristics including stage,Grade and age with patient survival in a given tumor sample
